# Organ- and Growing Stage-Specific Expression of Solanesol Biosynthesis Genes in *Nicotiana tabacum* Reveals Their Association with Solanesol Content

**DOI:** 10.3390/molecules21111536

**Published:** 2016-11-15

**Authors:** Ning Yan, Hongbo Zhang, Zhongfeng Zhang, John Shi, Michael P. Timko, Yongmei Du, Xinmin Liu, Yanhua Liu

**Affiliations:** 1 Tobacco Research Institute of Chinese Academy of Agricultural Sciences, Qingdao 266101, China; yanning@caas.cn (N.Y.); zhanghongbo@caas.cn (H.Z.); zhangzhongfeng@caas.cn (Z.Z.); duyongmei@caas.cn (Y.D.); liuxinmin@caas.cn (X.L.); 2 Guelph Food Research Center, Agriculture and Agri-Food Canada, Guelph, ON N1G 5C9, Canada; john.shi@agr.gc.ca; 3 Department of Biology, University of Virginia, Charlottesville, VA 22904, USA; mpt9g@virginia.edu

**Keywords:** *Nicotiana tabacum*, solanesol, RNA-seq, solanesyl diphosphate synthase, gene expression, chlorophyll

## Abstract

Solanesol is a noncyclic terpene alcohol that is composed of nine isoprene units and mainly accumulates in solanaceous plants, especially tobacco (*Nicotiana tabacum* L.). In the present study, RNA-seq analyses of tobacco leaves, stems, and roots were used to identify putative solanesol biosynthesis genes. Six 1-deoxy-d-xylulose 5-phosphate synthase (DXS), two 1-deoxy-d-xylulose 5-phosphate reductoisomerase (DXR), two 2-*C*-methyl-d-erythritol 4-phosphate cytidylyltransferase (*IspD*), four 4-diphosphocytidyl-2-*C*-methyl-d-erythritol kinase (*IspE*), two 2-*C*-methyl-d-erythritol 2,4-cyclo-diphosphate synthase (*IspF*), four 1-hydroxy-2-methyl-2-(*E*)-butenyl 4-diphosphate synthase (*IspG*), two 1-hydroxy-2-methyl-2-(*E*)-butenyl 4-diphosphate reductase (*IspH*), six isopentenyl diphosphate isomerase (*IPI*), and two solanesyl diphosphate synthase (SPS) candidate genes were identified in the solanesol biosynthetic pathway. Furthermore, the two *N. tabacum* SPS proteins (*NtSPS1* and *NtSPS2*), which possessed two conserved aspartate-rich DDxxD domains, were highly homologous with SPS enzymes from other solanaceous plant species. In addition, the solanesol contents of three organs and of leaves from four growing stages of tobacco plants corresponded with the distribution of chlorophyll. Our findings provide a comprehensive evaluation of the correlation between the expression of different biosynthesis genes and the accumulation of solanesol, thus providing valuable insight into the regulation of solanesol biosynthesis in tobacco.

## 1. Introduction

Solanesol is a medically important noncyclic terpene alcohol that is synthesized by the condensation of nine isoprene units. The molecule is a precursor of ubiquinone, also known as coenzyme Q_10_, vitamin K_2_, and anticancer drugs, including *N*-solanesyl-*N*,*N*′-bis(3,4-dimethoxybenzyl) ethylenediamine (SDB) [1,2,3]. Coenzyme Q_10_ possesses both antioxidant and antiaging properties and is reported to strengthen the body’s immune system and cardiovascular function, to improve brain health, and to moderate blood lipids. As a result, coenzyme Q_10_ has potential usefulness in the treatment of migraines, neurodegenerative diseases, hypertension, and cardiovascular diseases [2,3,4,5], and it is also being used as a dietary supplement by patients with type-2 diabetes [6]. Meanwhile, vitamin K_2_ promotes bone formation and mineralization, inhibits bone resorption, prevents and mitigates osteoporosis, promotes blood coagulation, and improves arterial stiffness [7]; and SDB, mediated by P-proteins, is used in the treatment of several types of drug resistance in tumours and plays a synergistic role with certain antitumor drugs [8,9]. Recently, Yao et al. [10] reported that solanesol can protect human hepatic L02 cells from ethanol-induced oxidative injury via upregulation of HO-1 and Hsp70 expression. Thus, the medical benefits of solanesol and its derivatives are well established.

**Scheme 1:**
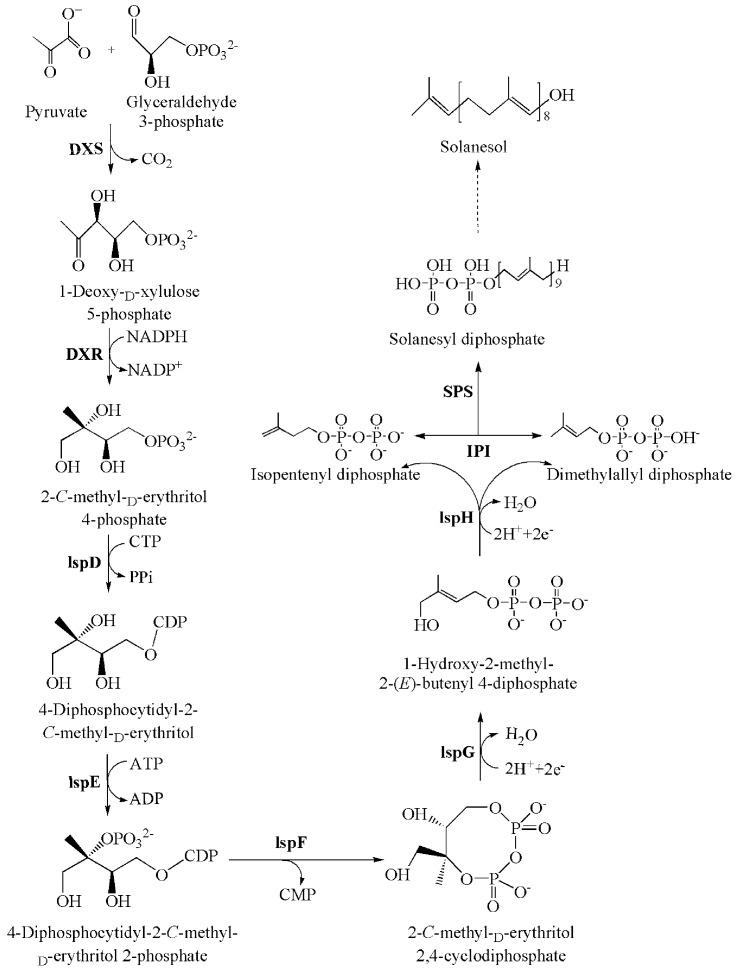
The solanesol biosynthetic pathway in the plastids of tobacco plants. Enzymes in the solanesol biosynthetic pathway are abbreviated as follows: DXS, 1-deoxy-d-xylulose 5-phosphate synthase; *DXR*, 1-deoxy-d-xylulose 5-phosphate reductoisomerase; IspD, 2-*C*-methyl-d-erythritol 4-phosphate cytidylyltransferase; IspE, 4-diphosphocytidyl-2-*C*-methyl-d-erythritol kinase; *IspF*, 2-*C*-methyl-d-erythritol 2,4-cyclodiphosphate synthase; *IspG*, 1-hydroxy-2-methyl-2-(*E*)-butenyl 4-diphosphate synthase; *IspH*, 1-hydroxy-2-methyl-2-(*E*)-butenyl 4-diphosphate reductase; IPI, isopentenyl diphosphate isomerase; SPS, solanesyl diphosphate synthase.

The molecule was first isolated from tobacco (*Nicotiana tabacum* L.) in 1956 and has subsequently been reported to occur in other solanaceous plants, including tomatoes, potatoes, eggplants, and peppers [1,3,11,12], in which it occurs in both free and ester-bound states [3,12,13]. Solanesol biosynthesis occurs in the plastids of higher plants via the 2-*C*-methyl-d-erythritol 4-phosphate (MEP) pathway [3,12,14], and Yan et al. [3] and Taylor and Fraser [12] have reported that the key enzymes of solanesol biosynthesis include 1-deoxy-d-xylulose 5-phosphate synthase (DXS), 1-deoxy-d-xylulose 5-phosphate reductoisomerase (*DXR*), 2-*C*-methyl-d-erythritol 4-phosphate cytidylyltransferase (*IspD*), 4-diphosphocytidyl-2-*C*-methyl-d-erythritol kinase (*IspE*), 2-*C*-methyl-d-erythritol 2,4-cyclodiphosphate synthase (*IspF*), 1-hydroxy-2-methyl-2-(*E*)-butenyl 4-diphosphate synthase (*IspG*), 1-hydroxy-2-methyl-2-(*E*)-butenyl 4-diphosphate reductase (*IspH*), isopentenyl diphosphate isomerase (IPI), and solanesyl diphosphate synthase (SPS) (Scheme 1) [3,12]. However, as a long-chain polyisoprenoid alcohol, solanesol is difficult to synthesize de novo [11], and solanesol is primarily produced via extraction from plants, particularly tobacco leaves [3,12].

Currently, nothing is known about the expression of genes involved in the biosynthetic pathway of solanesol in tobacco, and although some studies have investigated the solanesol content of tobacco leaves [3,12], little is known about the relative contents of different states of solanesol in different organs or at different growing stages. Therefore, in the present study, we investigated the expression of solanesol biosynthesis genes and the accumulation of solanesol in three organs (leaves, stems, and roots) of tobacco plants, as well as in the leaves of tobacco plants at four growing stages. This work provides a starting point for further research regarding solanesol biosynthesis genes in tobacco, as well as in other solanaceous plants, and provides valuable insight into the regulation of solanesol biosynthesis in tobacco.

## 2. Results

### 2.1. Organ- and Growing Stage-Specific Solanesol Content

Leaf tissues were harvested from four growing stages of tobacco plants (S1, S2, S3, and S4) at 10, 20, 40, and 60 days after transplanting, respectively; and root, stem, and leaf samples were harvested from S3-stage plants.

**Figure 1:**
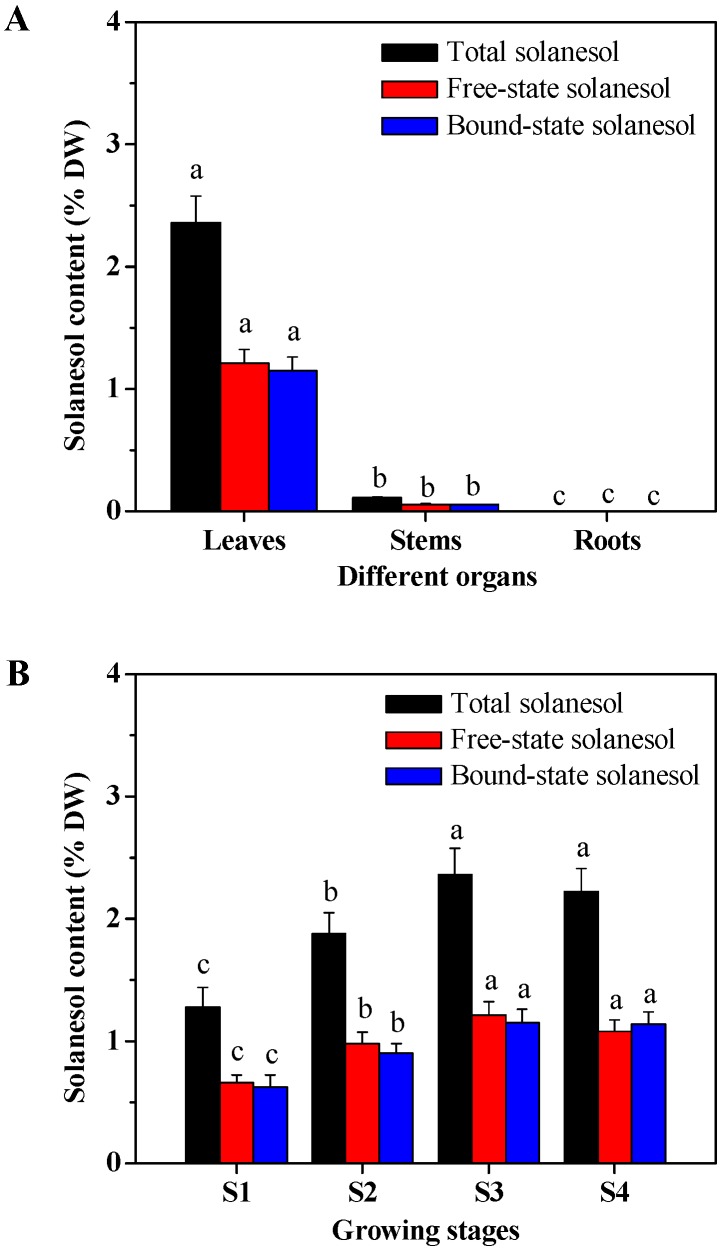
Solanesol content of tobacco plants. (**A**) Solanesol content of different organs of S3-stage tobacco plants; (**B**) Solanesol content of leaves harvested from four growing stages of tobacco plants. Values and error bars represent means ± SD. Different lowercase letters indicate significant differences (*p* < 0.05) between organs or growing stages. S1, 10 days after transplanting; S2, 20 days after transplanting; S3, 40 days after transplanting; S4, 60 days after transplanting; DW, dry weight.

The solanesol content was measured using ultra-high performance liquid chromatography (UPLC) method. The solanesol content was highest in the leaves of S3-stage tobacco plants, followed by that in the stems and roots (*p* < 0.05); and the levels of total, free-state, and bound-state solanesol in the leaves were 21.45-, 21.23-, and 21.70-fold that in the stems, respectively (Figure 1A), whereas no measurable solanesol was detected in the roots. The total, free-state, and bound-state solanesol content of leaves from the four growing stages of plants were significantly different, and the content was lowest at the S1 stage, increased at the S2 stage, reached a maximum at the S3 stage, and then decreased slightly at the S4 stage (Figure 1B).

### 2.2. Organ-Specific Expression of Solanesol Biosynthesis Genes

**Figure 2:**
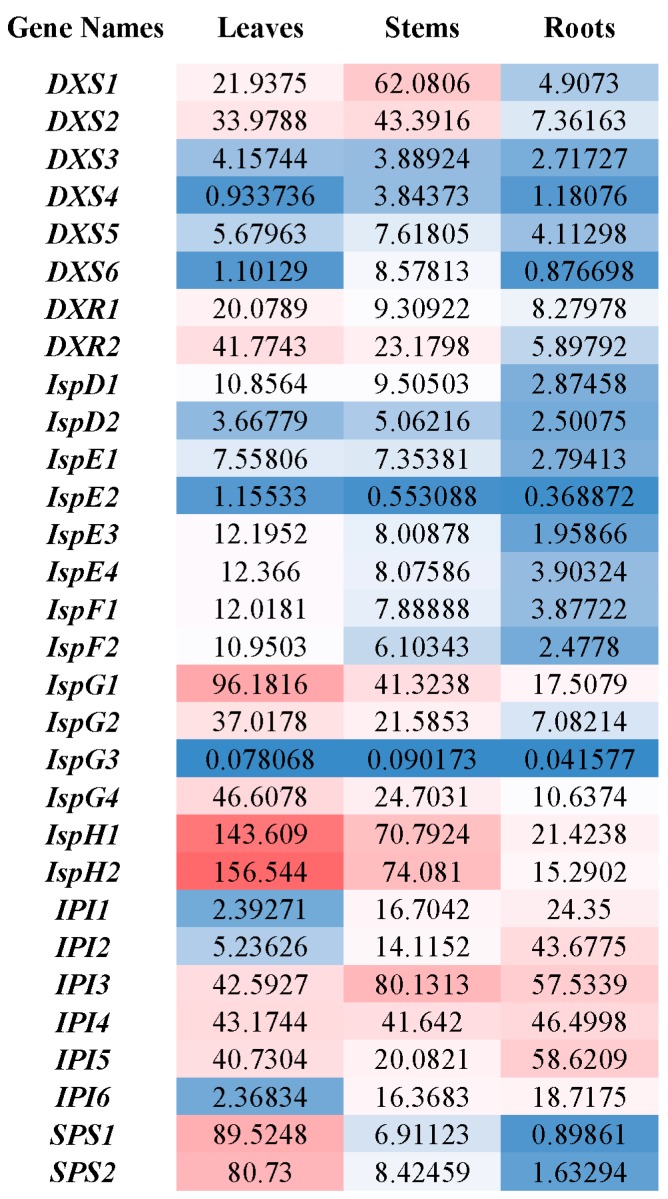
Expression heat map of solanesol biosynthesis genes in the leaves, stems, and roots of tobacco plants. The expression levels are expressed as fragments per kilobase of exon per million fragments mapped (FPKM) values. Red indicates high levels of expression, and blue indicates low levels of expression.

To identify candidate genes in the solanesol biosynthetic pathway, RNA-seq analyses of the leaves, stems, and roots of S3-stage tobacco plants were conducted. Six *DXS*, two *DXR*, two *IspD*, four *IspE*, two *IspF*, four *IspG*, two *IspH*, six IPI, and two SPS candidate genes were identified based on the similarity of the predicted amino acidic sequences to known sequences in the National Center for Biotechnology Information (NCBI) GenBank database (Figure 2). For gene expression analysis, the number of expressed sequence tags was calculated, and the numbers were normalized to fragments per kilobase of exon per million fragments mapped (FPKM) reads.

The FPKM values of most (~57%) of the genes (*DXS3*, *DXR1*, *DXR2*, *IspD1*, *IspE1*, *IspE2*, *IspE3*, *IspE4*, *IspF1*, *IspF2*, *IspG1*, *IspG2*, *IspG4*, *IspH1*, *IspH2*, *SPS1*, and *SPS2*) were highest in the leaves and lowest in the roots (leaves > stems > roots; *p* < 0.05; Figure 2). However, the FPKM values of several genes (*DXS1*, *DXS2*, *DXS5*, *DXS6*, *IspD2*, and *IspG3*) were highest in the stems (stems > leaves > roots), and other patterns were observed for *DXS4* and *IPI3* (stems > roots > leaves; *p* < 0.05); *IPI1*, *IPI2*, and *IPI6* (roots > stems > leaves; *p* < 0.05); and *IPI4* and *IPI5* (roots > leaves > stems; *p* < 0.05).

More specifically, the FPKM values of *DXS3*, *DXR1*, *DXR2*, *IspD1*, *IspE1*, *IspE2*, *IspE3*, *IspE4*, *IspF1*, *IspF2*, *IspG1*, *IspG2*, *IspG4*, *IspH1*, and *IspH2* ranged from 2% (*IspE1*) to 1195% (*SPS1*) higher in the leaves than in the stems and from 3% (*IspF1*) to 669% (*SPS1*) higher in the stems than in the roots (Figure 2). For *DXS1*, *DXS2*, *DXS5*, *DXS6*, *IspD2*, and *IspG3*, the FPKM values ranged from 16% (*IspG3*) to 679% (*DXS6*) higher in the stems than in the leaves and from 26% (*DXS6*) to 362% (*DXS2*) higher in the leaves than in the roots. For *DXS4* and *IPI3*, the FPKM values ranged from 39% (*IPI3*) to 226% (*DXS4*) higher in the stems than in the roots and from 26% (*DXS4*) to 35% (*IPI3*) higher in the roots than in the leaves. For *IPI1*, *IPI2*, and *IPI6*, the FPKM values ranged from 14% (*IPI6*) to 209% (*IPI6*) higher in the roots than in the stems and from 170% (*IPI2*) to 598% (*IPI1*) higher in the stems than in the leaves. For *IPI4* and *IPI5*, the FPKM values ranged from 8% (*IPI4*) to 44% (*IPI5*) higher in the roots than in the leaves and from 4% (*IPI4*) to 103% (*IPI5*) higher in the leaves than in the stems.

### 2.3. Organ- and Growing Stage-Specific NtSPS Expression

**Figure 3:**
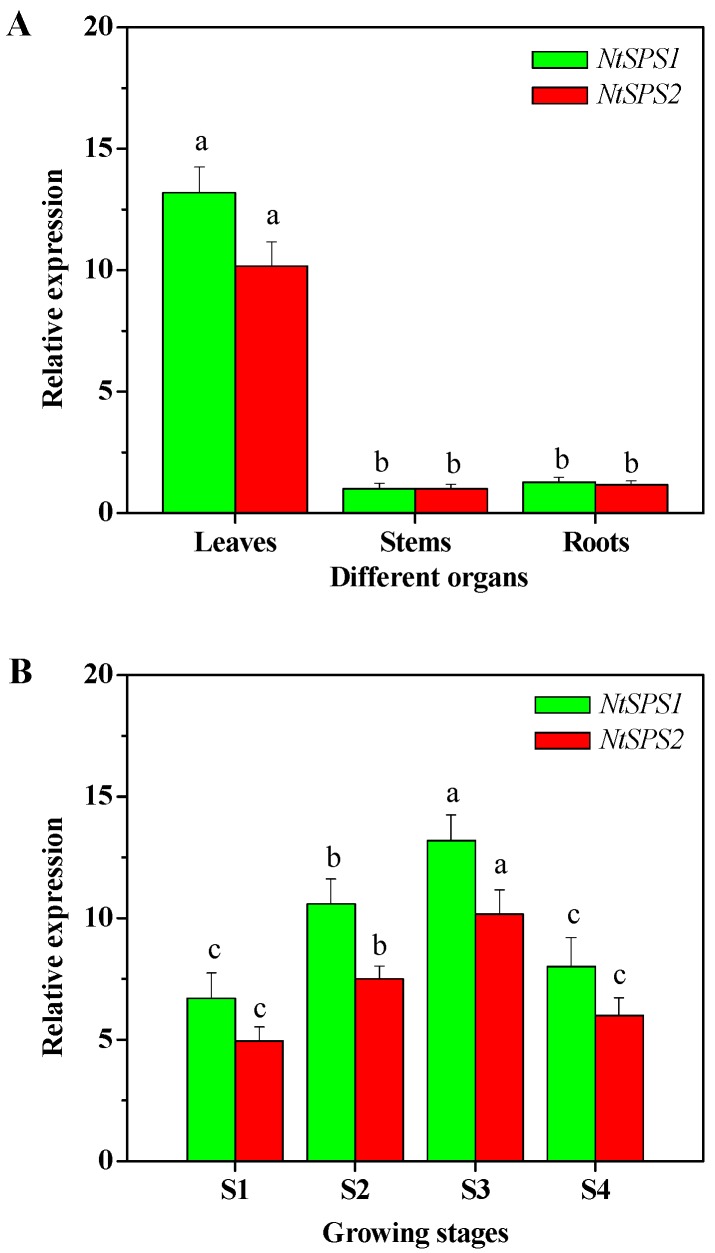
Relative expression of solanesyl diphosphate synthase (*NtSPS*) in tobacco plants. (**A**) *NtSPS* expression in different organs of S3-stage tobacco plants; (**B**) *NtSPS* expression in leaves harvested from four growing stages of tobacco plants. Values and error bars represent means ± SD. Different lowercase letters indicate significant differences (*p* < 0.05) between organs or growing stages. S1, 10 days after transplanting; S2, 20 days after transplanting; S3, 40 days after transplanting; S4, 60 days after transplanting.

The expression of two *N. tabacum SPS* genes (*NtSPS1* and *NtSPS2*) was analysed, in order to validate the results from RNA-seq analyses. The relative expression of the *NtSPS* genes was significantly higher in the leaves of the tobacco plants than in the stems and roots (*p* < 0.05), in which the *NtSPS* levels were statistically similar (*p* > 0.05) (Figure 3A), and the relative expression of *NtSPS1* and *NtSPS2* in the leaves was 13.19 and 10.17 fold that in the stems, respectively.

In addition, the relative expression of *NtSPS1* and *NtSPS2* also differed significantly among the leaves from the four growing stages. The expression was lowest in the leaves from S1-stage plants, intermediate in leaves from S2-stage plants, greatest in the leaves from S3-stage plants, and low again in the leaves from S4-stage plants (Figure 3B). Therefore, the relative expression of *NtSPS1* and *NtSPS2* was consistent with the observed solanesol contents.

### 2.4. Phylogenetic Analysis of NtSPS

**Figure 4:**
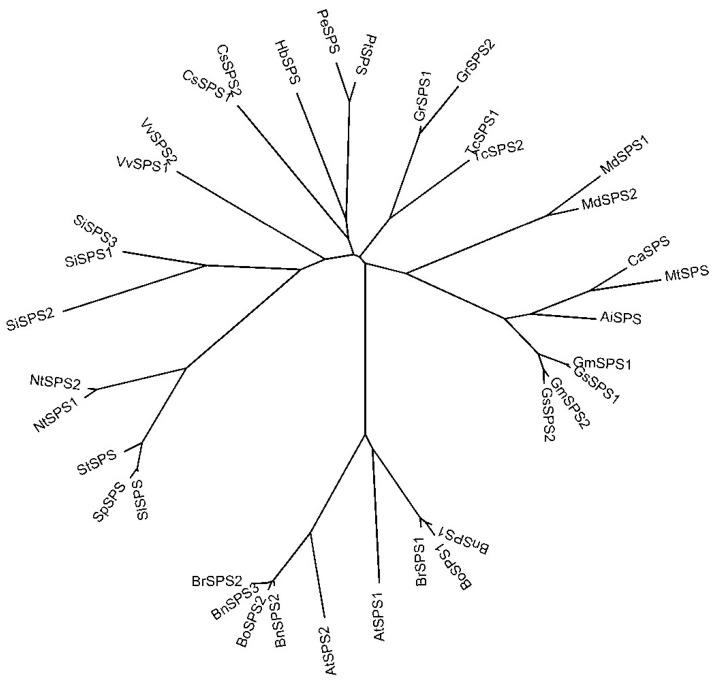
Molecular phylogenetic tree of solanesyl diphosphate synthase (SPS) amino acid sequences from different plant species. The tree was constructed using the neighbour-joining method in MEGA 5.0. The enzymes used for alignment are as follows: AiSPS (*A. ipaensis*, XP_016171459.1), AtSPS1 (*A. thaliana*, ABF58968.1), AtSPS2 (*A. thaliana*, NP_173148.2), BnSPS1 (*B. napus*, XP_013649976.1), BnSPS2 (*B. napus*, XP_013660438.1), BnSPS3 (*B. napus*, XP_013641601.1), BoSPS1 (*B. oleracea* var. oleracea, XP_013592833.1), BoSPS2 (*B. oleracea* var. oleracea, XP_013637933.1), BrSPS1 (*B. rapa*, XP_009106560.1), BrSPS2 (*B. rapa*, XP_009149144.1), CaSPS (*C. arietinum*, XP_004488673.1), CsSPS1 (*C. sinensis*, XP_015385554.1), CsSPS2 (*C. sinensis*, XP_015385553.1), GmSPS1 (*G. max*, XP_003543173.1), GmSPS2 (*G. max*, XP_003546746.1), GrSPS1 (*G. raimondii*, XP_012462942.1), GrSPS2 (*G. raimondii*, XP_012462943.1), GsSPS1 (*G. soja*, KHN47455.1), GsSPS2 (*G. soja*, KHN39753.1), HbSPS (*H. brasiliensis*, ABD92707.1), MdSPS1 (*M. domestica*, XP_008363649.1), MdSPS2 (*M. domestica*, XP_008386386.1), MtSPS (*M. truncatula*, XP_013464447.1), PeSPS (*P. euphratica*, XP_011045575.1), PtSPS (*P. trichocarpa*, XP_002300442.2), SiSPS1 (*S. indicum*, XP_011098150.1), SiSPS2 (*S. indicum*, XP_011082816.1), SiSPS3 (*S. indicum*, XP_011098151.1), SlSPS (*S. lycopersicum*, XP_004244308.1), SpSPS (*S. pennellii*, XP_015082582.1), StSPS (*S. tuberosum*, XP_006348350.1), TcSPS1 (*T. cacao*, XP_007022091.1), TcSPS2 (*T. cacao*, XP_007022090.1), VvSPS1 (*V. vinifera*, XP_002285665.1), and VvSPS2 (*V. vinifera*, XP_010644050.1).

**Figure 5:**
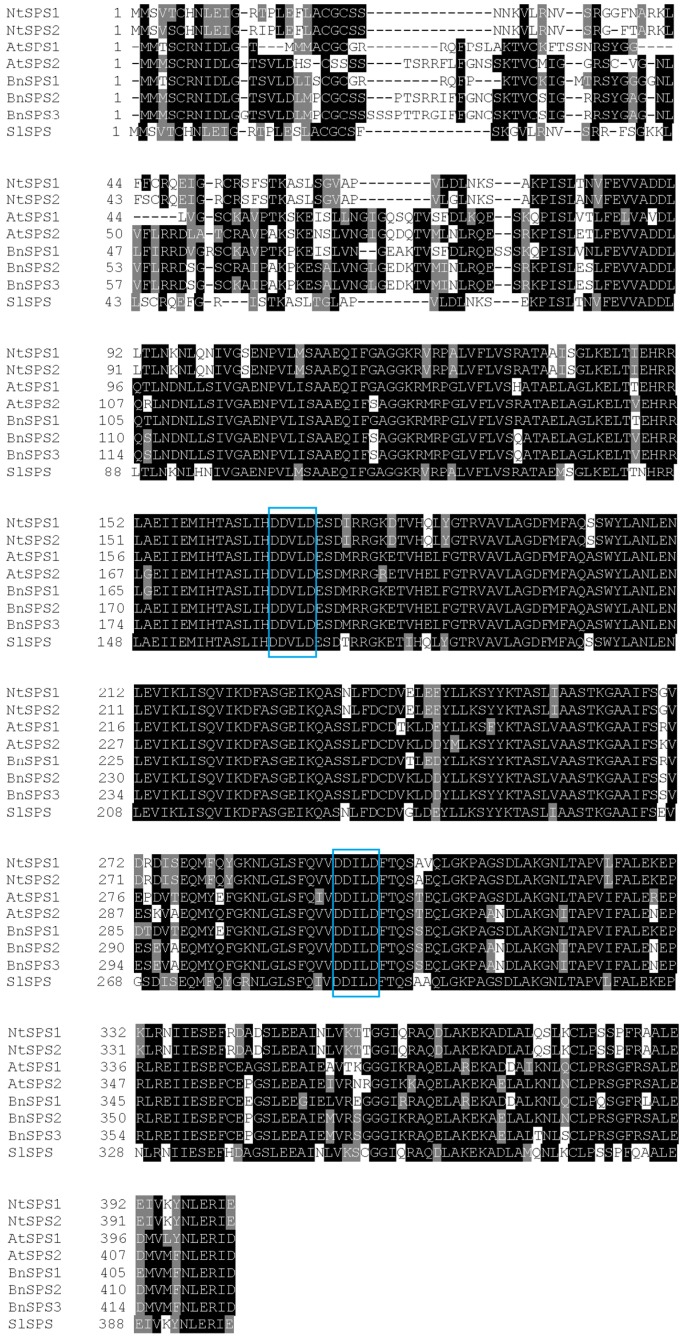
Multiple sequence alignment of solanesyl diphosphate synthase (SPS) amino acid sequences from tobacco (*N. tabacum*), tomato (*L. esculentum*), *A. thaliana*, and *B. napus*. The conserved functional motif, DDxxD, is boxed in blue. Identical and similar amino acid residues are shaded in black and grey, respectively. The alignment of full-length predicted amino acid sequences was performed using MultiAlign and shaded using BOXSHADE 3.21 (http://www.ch.embnet.org/software/BOX_form.html). AtSPS1 (*A. thaliana*, ABF58968.1), AtSPS2 (*A. thaliana*, NP_173148.2), BnSPS1 (*B. napus*, XP_013649976.1), BnSPS2 (*B. napus*, XP_013660438.1), BnSPS3 (*B. napus*, XP_013641601.1), and SlSPS (*S. lycopersicum*, XP_004244308.1).

To define the phylogenetic relationships among the SPS proteins from tobacco (*NtSPS1* and *NtSPS2*) and other plant species, we downloaded 37 plant SPS sequences from the NCBI database and used these data to construct a phylogenetic tree (Figure 4). In the phylogenetic analysis, the *NtSPS1* and *NtSPS2* sequences clustered with those from other solanaceous plants, i.e., SlSPS from *Solanum lycopersicum*, SpSPS from *S. pennellii*, and StSPS from *S. tuberosum* (Figure 4), which suggests that the biological function of the tobacco SPS proteins is similar to that reported for other solanaceous plants. Similarly, the SPS sequences from brassicaceous plants (e.g., *Arabidopsis thaliana*, *B. napus*, *B. oleracea* var. oleracea, and *B. rapa*) also formed a distinct cluster (Figure 4). In addition, a multiple sequence alignment of the SPS amino acid sequences from *A. thaliana*, *B. napus*, tomato, and tobacco revealed the presence of two conserved aspartate-rich DDxxD domains (Figure 5).

### 2.5. Organ- and Growing Stage-Specific Chlorophyll Content

**Figure 6:**
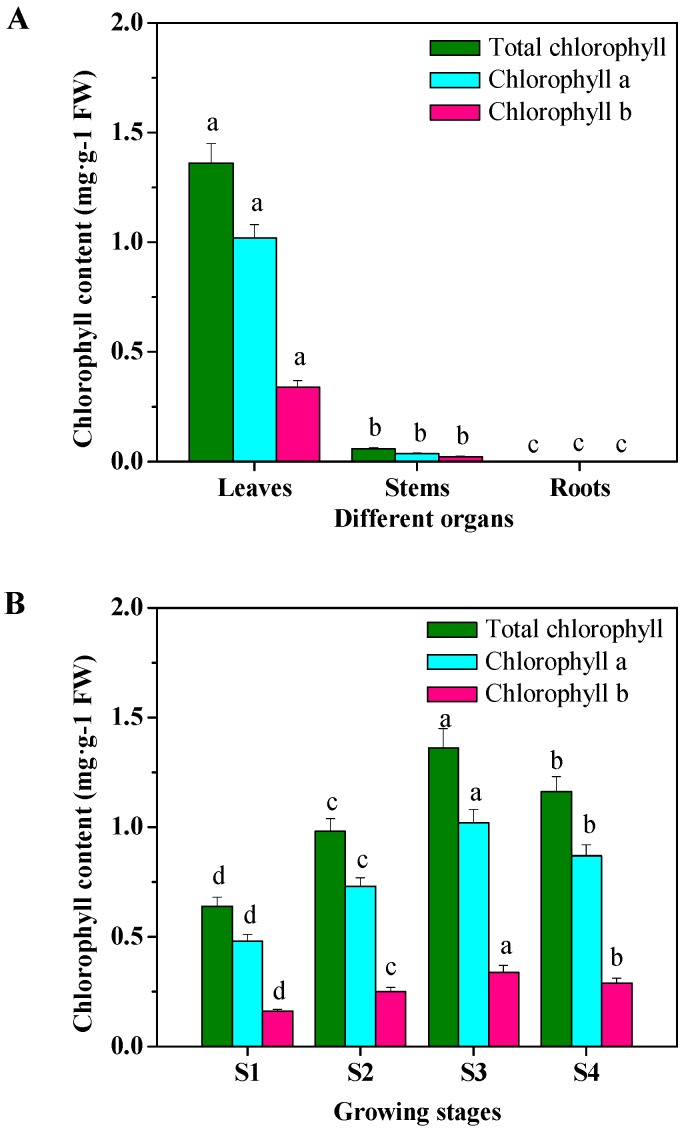
Chlorophyll content of tobacco plants. (**A**) Chlorophyll content of different organs of S3-stage tobacco plants; (**B**) Chlorophyll content of leaves harvested from four growing stages of tobacco plants. Values and error bars represent means ± SD. Different lowercase letters indicate significant differences (*p* < 0.05) between organs or growing stages. S1, 10 days after transplanting; S2, 20 days after transplanting; S3, 40 days after transplanting; S4, 60 days after transplanting; FW, fresh weight.

The contents of total chlorophyll, chlorophyll a, and chlorophyll b were highest in the leaves of the S3-stage tobacco plants, followed by the levels detected in the stems and roots, respectively (*p* < 0.05) (Figure 6A). In the leaves, the content of total chlorophyll, chlorophyll a, and chlorophyll b were 23.05-, 28.33-, and 14.78-fold that observed in the stems, respectively (Figure 6A), and no chlorophyll was detected in the roots. Significant differences in chlorophyll content were also observed in the leaves collected from the four growing stages, and all parameters were lowest in the leaves from S1-stage plants, intermediate in leaves from S2-stage plants, greatest in the leaves from S3-stage plants, and slightly lower than the observed maximum in the leaves from S4-stage plants (Figure 6B). These changes were consistent with the distribution of solanesol in the three organs and the levels of solanesol detected at four growing stages.

## 3. Discussion

### 3.1. Solanesol Content of Tobacco Plants

Solanesol is a long-chain polyisoprenoid alcohol that mainly accumulates in solanaceous plants, especially tobacco [1,3,12], and is an important intermediate in the synthesis of ubiquinone and anti-cancer drugs. Because the chemical synthesis of solanesol is difficult [11], we assessed some aspects of its biosynthesis in tobacco plants and observed the differential accumulation of solanesol in the leaves, stems, and roots of tobacco plants, with the content in leaves being the highest (Figure 1A). Our results also revealed that the chlorophyll content in the leaves, stems, and roots of tobacco plants (Figure 6A) corresponded with the distribution of solanesol (Figure 1A), which is consistent with other studies that have suggested that solanesol is synthesized in chloroplasts [3,12,14], and our results also indicate that solanesol mainly accumulates in green plant tissues (e.g., leaves and stems). Thus, tobacco leaves are likely an ideal source material for solanesol extraction [3,12].

The results of the present study also indicated that the solanesol content of leaves varies as the plants develop, and the solanesol content was highest in leaves from S3-stage plants (40 days after transplanting; Figure 1B). Therefore, the S3 stage can be considered the most appropriate period during which to harvest tobacco leaves for solanesol extraction. However, since the accumulation of solanesol is reportedly influenced by genetic and environmental factors [1,3,12,15], the optimal harvest period for solanesol extraction may vary, depending on individual tobacco varieties and environmental conditions.

### 3.2. Solanesol Biosynthesis Genes in Tobacco

Based on the similarity of amino acid sequences, six *DXS*, two *DXR*, two *IspD*, four *IspE*, two *IspF*, four *IspG*, two *IspH*, six *IPI*, and two *SPS* candidate genes were identified in the solanesol biosynthetic pathway of tobacco (Figure 2).

DXS is the first enzyme in the MEP pathway and is responsible for catalysing the conversion of pyruvate and glyceraldehyde 3-phosphate to form 1-deoxy-d-xylulose 5-phosphate (DXP) (Scheme 1) [3,12]. In the present study, we assessed the FPKM values of different *DXS* genes in the leaves, stems, and roots of tobacco plants, and the relative expression levels of *DXS1*, *DXS2*, *DXS3*, *DXS5*, and *DXS6* were higher in the tobacco leaves and stems than in the roots (Figure 2). In *Medicago truncatula*, *MtDXS1* was preferentially expressed in several aboveground tissues (e.g., leaves and stems) but not in the roots, whereas *MtDXS2* transcript levels were low in most tissues but strongly stimulated in roots upon colonization by mycorrhizal fungi [16]. Compared with non-transgenic wild-type plants, transgenic *Arabidopsis* plants that over- or underexpressed *DXS* gene accumulated different levels of various isoprenoids, including chlorophylls, tocopherols, carotenoids, abscisic acid, and gibberellins [17]; and when the *A. thaliana DXS* gene was constitutively expressed in spike lavender, the leaves of flowers of the transgenic plants accumulated significantly more essential oils (monoterpenes) [18]. Thus, as an important regulatory factor in the MEP pathway, *DXS* is a crucial enzyme in the solanesol biosynthetic pathway, and its overexpression or inhibition can influence the content of downstream metabolites.

Meanwhile, *DXR* catalyses the intramolecular rearrangement of DXP’s straight-chain carbon skeleton to MEP (Scheme 1) [3,12]. In the present study, the variation in FPKM values of *DXR1* and *DXR2* in leaves, stems, and roots (Figure 2) was consistent with the distribution of solanesol (Figure 1). Zhang et al. [19] cloned two *DXR* genes from *N. tabacum* and found that the expression of *NtDXR1* and *NtDXR2* was highest in tobacco leaves, followed by that in the stem and roots. In addition, the downregulation of *DXR* in *A. thaliana* results in variegation, reduced pigmentation, and defects in chloroplast development, whereas *DXR*-overexpressing lines exhibit increased accumulation of MEP-derived plastid isoprenoids, such as chlorophylls and carotenoids [20]. In contrast, the overexpression of the tobacco *DXR* gene in chloroplasts has been shown to increase the production of isoprenoids, including solanesol, chlorophyll a, beta-carotene, lutein, antheraxanthin, and beta-sitosterol [21]. Therefore, *DXR* is obviously another key enzyme in the biosynthesis of solanesol in tobacco, and its overexpression in chloroplasts can promote the accumulation of solanesol.

*IspD*, *IspE*, *IspF*, *IspG*, and *IspH* sequentially catalyse the transformation of MEP to IPP and DMAPP (Scheme 1) [3,22]. In the present study, we observed variation in the FPKM values of Isp genes in leaves, stems, and roots (Figure 2), and the relative expression levels of *IspE*, *IspF*, *IspG*, and *IspH* were higher in the tobacco leaves and stems than in the roots. Similarly, Hsieh et al. [23] observed that *IspD1*, *IspD2*, and *IspE1* are mainly expressed in the leaves and stems of *A. thaliana*. However, Kim et al. [24] cloned an *IspF* gene from *Ginkgo biloba* and found that its expression was higher in embryonic roots than in embryonic leaves. Gao et al. [25] also reported that the expression of *IspF* was highest in *G. biloba* roots, followed by that in the leaves and seeds, respectively, and Lu et al. [26] cloned the *IspH* gene from *G. biloba* and found that its expression was highest in roots, as well, followed by its expression in stems and leaves. In contrast, Kim et al. [27] cloned two *IspH* genes from *G. biloba* and found that the expression of *IspH1* was higher in the leaves than in the roots, whereas the expression of *IspH2* was higher in the roots than in the leaves. Thus, our results regarding the distribution of *IspD*, *IspE*, *IspF, IspG*, and *IspH* expression are not fully consistent with those of previous studies in other plants, and further investigation is warranted.

*IPI* catalyses the isomerization of IPP and DMAPP, which requires Mg^2+^, and the resulting IPP and DMAPP can then bind to form isoprenoids, such as solanesol (Scheme 1) [3,28]. In the present study, we assessed the FPKM values of different *IPI* genes in the leaves, stems, and roots of tobacco plants, and the relative expression levels of *IPI1*, *IPI2*, *IPI4*, *IPI5*, and *IPI6* were highest in tobacco roots of these three organs (Figure 2). Nakamura et al. [29] cloned two *N. tabacum IPI* genes and found that the expression of *IPI1* increases under high-salt and high-light stress conditions, whereas the expression of *IPI2* increases under high-salt and cold stress conditions. Sun et al. [30] cloned an *IPI* gene from *S. lycopersicum* and found that its expression was highest in roots, followed by that in stems and leaves, which was consistent with the distribution of *IPI1*, *IPI2*, and *IPI6* expression observed in the present study. Thus, our results regarding the distribution of different *IPI* genes expression are not fully consistent with those of previous studies, and further investigation is warranted.

SPS catalyses the reaction of IPP and DMAPP to form SPP, which is a precursor of solanesol (Scheme 1) [3,12]. In the present study, both qRT-PCR and RNA-seq analyses indicated that the relative expression of *NtSPS1* and *NtSPS2* were significantly higher in the leaves than in the stems and roots (*p* < 0.05) (Figure 2 and Figure 3). To date, *SPS* homologs have been identified in *A. thaliana* [31,32,33], *Hevea brasiliensis* [34], *Oryza sativa* [35], and *S. lycopersicum* [36]. Hirooka et al. [32] cloned two *SPS* genes from *A. thaliana* and found that the expression of *AtSPS1* and *AtSPS2* was significantly higher in stems and leaves than in roots, which is similar to the expression of *NtSPS1* and *NtSPS2* observed in the present study (Figure 3). The homology between *NtSPS1* and *NtSPS2* and SPS proteins from other solanaceous plants was relatively higher than that in other families (Figure 4), and our results suggest that the biological function of SPS proteins in solanaceous plants is similar to that reported in other plants [37,38]. SPS proteins from other plants contain two conserved aspartate-rich DDxxD domains (Figure 5), which are involved in the coordination of divalent metal ions with the diphosphate groups in substrates and play a key role in substrate positioning [39]. Jones et al. [36] suggested that the constitutive overexpression of *SlSPS* in tobacco could significantly increase the solanesol content of mature leaves, and also reported that the solanesol content of mature leaves from transgenic tobacco plants was positively correlated with the expression of *SlSPS*. Thus, SPS is a key enzyme in the solanesol biosynthetic pathway, and its overexpression can promote the accumulation of downstream metabolites, such as solanesol [3,12,36].

## 4. Experimental Section

### 4.1. Plant Materials and Growing Conditions

*N. tabacum* (Honghua Dajinyuan) plants were used for RNA-seq analysis. The seed was obtained from China Tobacco Germplasm Platform (Qingdao, China), and the seedlings, which sprouted on 10 February 2014, were transplanted on 20 April 2014. The plants were grown and maintained in plastic containers filled with soil (pH: 7.2, total N: 1.89 g/kg, alkali-soluble N: 48.3 mg/kg, total P 0.45 g/kg, available P: 32.4 mg/kg, total K: 32.5 g/kg, available K: 219 mg/kg, organic matter: 7.39 g/kg) in a greenhouse at the Tobacco Research Institute of Chinese Academy of Agricultural Sciences (Qingdao, China) under natural conditions (16 h light at 28 °C during the day, 8 h dark at 23 °C during the night). To measure the solanesol and chlorophyll contents and to perform RNA-seq and quantitative real-time PCR (qRT-PCR) analyses, leaves were harvested from four growing stages (S1, S2, S3, and S4) of tobacco plants at 10, 20, 40, and 60 days after transplanting, respectively; and root, stem, and leaf samples were harvested from S3-stage plants. All the experiments were performed in triplicate.

### 4.2. Analysis of Total, Free-State, and Bound-State Solanesol Contents

The tobacco leaves, stems, and roots were dried to constant weight with a freeze-dryer (Alpha 1–2 LD Plus; Christ, Osterode am Harz, Germany), ground, and sifted through a 40-mesh sieve. Portions (2 g) of the powdered samples were transferred to individual 50-mL centrifuge tubes with stoppers, and 20 mL hexane was added. Ultrasonic extraction was performed at 65 °C for 15 min, and the resulting homogenates were centrifuged at 3000 rpm for 10 min, after which the supernatants were transferred to 50-mL volumetric flasks. The extraction steps were then repeated by extracting the precipitated layers two additional times, each with 15 mL hexane, and the two additional extracts for each sample were combined with the corresponding initial extracts.

To prepare the samples for quantification of free-state solanesol, 4 mL aliquots of the hexane extracts were transferred to individual 10-mL stoppered centrifuge tubes and supplemented with 6 mL distilled water. The mixtures were vortexed for 3 min to remove the water-soluble impurities and then centrifuged at 3000 rpm for 10 min. Next, the upper layers were removed, diluted with a 50:50 (*v*:*v*) methanol-acetonitrile solution in brown volumetric flasks, and then filtered through a 0.2-μm membrane.

Meanwhile, to prepare the samples for quantification of total solanesol, 4 mL aliquots of the hexane extracts were transferred to individual 100-mL brown stoppered flasks and supplemented with 4 mL 0.02 M NaOH (diluted in ethanol). After thorough mixing, the samples were oscillated in a water bath for 30 min at 60–65 °C to allow saponification and then incubated in an 83–87 °C water bath to allow the solvent to evaporate. Subsequently, 2 mL hexane was added to each sample, and the mixtures were subject to ultrasonication for 2 min, in order to dissolve the residue; transferred to clean 10-mL stoppered centrifuge tubes; and dissolved with another 2 mL hexane. Next, 6 mL distilled water was added, and the mixtures were vortexed for 3 min to remove the water-soluble impurities. After a 10-min centrifugation at 3000 rpm, the upper layers were removed, diluted with a 50:50 (*v*:*v*) methanol-acetonitrile solution in brown volumetric flasks, and then filtered through a 0.2-μm membrane.

The amounts of total and free-state solanesol were measured using ultra-high performance liquid chromatography (ACQUITY UPLC H-Class; Waters, Milford, MA, USA) with an Atlantis T3-C_18_ column (4.6 mm × 150 mm, 3 μm; Waters) that was maintained at 35 °C. A 50:50 (*v*:*v*) methanol-acetonitrile solution was used as the mobile phase at a flow rate of 1.0 mL/min, and a diode array detector was used for detection at 213 nm. Meanwhile, the amount of bound-state solanesol in each extract was calculated as the difference between the free-state and total solanesol levels.

### 4.3. Preparation of Digital Gene Expression Library, Sequencing, and Analysis

The roots, stems, and leaves collected from S3-stage tobacco plants were used to generate three digital gene expression libraries, in order to identify the genes in the solanesol biosynthetic pathway in tobacco plants, and plants with possible microbial contamination were excluded. Total RNA was extracted using TRIzol (Invitrogen, Carlsbad, CA, USA), and RNA degradation and contamination were assessed using electrophoresis on 1% agarose gels. The purity of the extracted RNA was checked spectrophotometrically, using a NanoPhotometer (Implen, Inc., Westlake Village, CA, USA), and the RNA was quantified using the Qubit RNA Assay Kit and a Qubit 2.0 fluorometer (Life Technologies, Carlsbad, CA, USA). The integrity of the RNA was assessed using an RNA Nano 6000 Assay Kit and the BioAnalyzer 2100 system (Agilent Technologies, Santa Clara, CA, USA).

Three mixtures that contained equal amounts of RNA from the three organs were prepared for each sample and subsequently used to construct the library. The library quality was assessed using the Agilent Bioanalyzer 2100 system (Agilent Technologies), and the clustering of index-coded samples was performed on a cBot Cluster Generation System using the TruSeq PE Cluster Kit v3-cBot-HS (Illumina, Santiago, CA, USA), according to the manufacturer’s instructions. After cluster generation, the library preparations were sequenced using an Illumina HiSeq platform to generate paired-end reads.

Dirty raw reads (i.e., reads with adapters, unknown nucleotides, or quality values ≤5 that accounted for >50% of the read) were discarded, since they would negatively affect downstream analyses, and de novo assembly of the short reads was performed using the Trinity assembly program, following the method described by Grabherr et al. [40]. Gene functions were identified by BLASTing the unigene sequences against protein databases, including the NCBI non-redundant (Nr) database (http://www.ncbi.nlm.nih.gov), Universal Protein Resource (UniProt) database (http://www.uniprot.org), and Cluster of Orthologous Groups of proteins (COG) database (http://www.ncbi.nlm.nih.gov/COG). For gene expression analysis, the number of expressed sequence tags was calculated, and the numbers were normalized to FPKM reads, according to the method of Mortazavi et al. [41]. The annotations and FPKM values of the unigenes were obtained from the China Tobacco Genome (CTG) database (http://218.28.140.17/).

### 4.4. Screening and Cloning of NtSPS1 and NtSPS2

In order to clone the two *NtSPS* genes, total RNA was isolated from tobacco leaves, and its quality was assessed as described in Section 4.3 above. In order to obtain first strand cDNA, reverse transcription was performed using a PrimerScript RT-PCR kit (Takara Bio, Inc., Shiga, Japan), and the cDNA was used as a template for PCR amplification, using gene-specific primers to amplify the complete coding sequences of both *NtSPS1* (upstream primer: 5′-ATGATGTCTGTGACTTGCCATAATC-3′, downstream primer: 5′-CTATTCAATTCTCTCCAGATTATACTTCAC-3′) and *NtSPS2* (upstream primer: 5′-ATGATGTCTGTGAGTTGCCATAATC-3′, downstream primer 5′-CTATTCAATTCTCTCCAGATTATACTTCAC-3′). The PCR amplification was performed as described by Block et al. [31], and the amplified products were gel extracted and transformed into competent *Escherichia coli* DH5α cells. Finally, the positive clones were screened and sequenced at the Beijing Genomics Institute (Shenzhen, China).

### 4.5. RNA Extraction, CDNA Synthesis, and QRT-PCR Analysis

Total RNA was extracted from the roots, stems, and leaves of S3-stage tobacco plants, as well as from the leaves of S1-, S2-, and S4-stage plants, as described above. Two micrograms of total extracted RNA from each sample was reverse transcribed to generate first-strand cDNA, using the PrimerScript kit (Takara Bio, Inc.) according to the manufacturer’s instructions, and the experiments were performed in triplicate. Gene-specific primer pairs were designed for sequence analysis of *NtSPS1* (upstream primer: 5′-TGTCTGTGACTTGCCATAA-3′, downstream primer 5′-CATTGAATCCTCC TCTACTT-3′) and *NtSPS2* (upstream primer: 5′-CAGTGTTGGGTTTGAATA-3′, downstream primer: 5′-CTTGTTTAGAGTAAGGAGGTC-3′), using the Primerquest software (http://www.idtdna.com/ pages/scitools), and qRT-PCR was performed using an ABI 7500 Real-time system (Applied Biosystems, Foster City, CA, USA) with SYBR Premix Ex Taq™ Kit (TaKaRa Bio, Inc.), according to the manufacturer’s protocol, and with the following amplification conditions: 95 °C for 2 min, followed by 40 cycles of 95 °C for 15 s and 60 °C for 1 min, and plate reading after each cycle. Two fragments of the constitutively expressed *Ntactin* gene were amplified as a reference, using the gene-specific upstream and downstream primers 5′-CATTCCAAATATGAGATGCGTTGT-3′ and 5′-TGTGGACTTGGGAGAGGACT-3′, respectively.

### 4.6. Phylogenetic Analysis of NtSPS

We aligned SPS amino acid sequences from different plant species using CLUSTAL W, computed the evolutionary distances between the sequences using the Poisson correction method, and constructed a neighbour-joining (NJ) tree using MEGA 5.0 [38]. The reliability of the topology was assessed using the bootstrap re-sampling method with 1000 bootstrap replications.

### 4.7. Determination of Chlorophyll Content

Chlorophyll was extracted from leaf, stem, and root samples by grinding 0.5 g of each sample in 1 mL of 100% acetone with a pinch of calcium carbonate in a mortar. The individual extracts were transferred to test tubes, after which the mortars were rinsed with 100% acetone, the rinsates were added to the extracts, and each sample extract was further diluted to 5 mL with acetone. Afterward, each of the extracts was filtered through a 0.45-μm syringe filter to remove the debris, and the absorbance of the filtered extracts at 663 nm (A_663_) and 645 nm (A_645_) was determined using a UV-2410PC spectrophotometer (Shimadzu, Tokyo, Japan). Then the chlorophyll a and chlorophyll b contents were calculated using the following equations: C_A_ = 0.25 × (12.7 × (A_663_) − 2.69 × (A_645_)) and C_B_ = 0.25 × (22.9 × (A_645_) − 4.68 × (A_663_)), where, C_A_ and C_B_ are the contents (mg/g) of chlorophyll a and b, respectively [42], and the total chlorophyll content was calculated as the sum of the chlorophyll a and chlorophyll b contents.

### 4.8. Accession Numbers

Sequence data for the genes described in this study can be found in the CTG database under the accession numbers shown in parentheses: *DXS1* (Ntab0104070), *DXS2* (Ntab0137200), *DXS3* (Ntab0429490), *DXS4* (Ntab0604300), *DXS5* (Ntab0667680), *DXS6* (Ntab0863750), *DXR1* (Ntab0057390), *DXR2* (Ntab0119750), *IspD1* (Ntab0014700), *IspD2* (Ntab0479870), *IspE1* (Ntab0204750), *IspE2* (Ntab0204760), *IspE3* (Ntab0786730), *IspE4* (Ntab0826890), *IspF1* (Ntab0883640), *IspF2* (Ntab0907560), *IspG1* (Ntab0346180), *IspG2* (Ntab0578920), *IspG3* (Ntab0763840), *IspG4* (Ntab0829890), *IspH1* (Ntab0167890), *IspH2* (Ntab0212660), *IPI1* (Ntab0340070), *IPI2* (Ntab0353610), *IPI3* (Ntab0520040), *IPI4* (Ntab0881480), *IPI5* (Ntab0901260), *IPI6* (Ntab0958550), *SPS1* (Ntab0214390), *SPS2* (Ntab0720120).

### 4.9. Statistical Analysis

The data were analysed using analysis of variance (ANOVA) with SPSS 18.0 statistical software (SPSS, Inc., Chicago, IL, USA), and significant differences between the treatments were investigated using Tukey’s multiple comparison test at the *p* < 0.05 significance level.

## 5. Conclusions

The present study demonstrates that solanesol is abundant in tobacco leaves and provides a starting point for further research regarding solanesol biosynthesis genes in tobacco plants. Six DXS, two *DXR*, two *IspD*, four *IspE*, two *IspF*, four *IspG*, two *IspH*, six *IPI*, and two SPS candidate genes were identified in the solanesol biosynthetic pathway of tobacco, and the two *N. tabacum* SPS proteins (*NtSPS1* and *NtSPS2*), which possessed two conserved aspartate-rich DDxxD domains, were highly homologous with the SPS enzymes of other solanaceous plant species. In addition, the solanesol contents of three organs (leaves, stems, and roots) and leaves from four growing stages of tobacco plants corresponded with the distribution of chlorophyll. Our findings provide a comprehensive evaluation of the correlation between the expression of different biosynthesis genes and the accumulation of solanesol, thus providing valuable insight into the regulation of solanesol biosynthesis in tobacco.

## References

[1] Campbell R., Freitag S., Bryan G.J., Stewart D., Taylor M.A. (2016). Environmental and genetic factors associated with solanesol accumulation in potato leaves. Front. Plant Sci..

[2] Parmar S.S., Jaiwal A., Dhankher O.P., Jaiwal P.K. (2015). Coenzyme Q_10_ production in plants: Current status and future prospects. Crit. Rev. Biotechnol..

[3] Yan N., Liu Y., Gong D., Du Y., Zhang H., Zhang Z. (2015). Solanesol: A review of its resources, derivatives, bioactivities, medicinal applications, and biosynthesis. Phytochem. Rev..

[4] Bentinger M., Tekle M., Dallner G. (2010). Coenzyme Q-Biosynthesis and functions. Biochem. Biophys. Res. Commun..

[5] Sarmiento A., Diaz-Castro J., Pulido-Moran M., Kajarabille N., Guisado R., Ochoa J.J. (2016). Coenzyme Q_10_ supplementation and exercise in healthy humans: A systematic review. Curr. Drug Metab..

[6] Mezawa M., Takemoto M., Onishi S., Ishibashi R., Ishikawa T., Yamaga M., Fujimoto M., Okabe E., He P., Kobayashi K. (2012). The reduced form of coenzyme Q_10_ improves glycemic control in patients with type 2 diabetes: An open label pilot study. BioFactors.

[7] Hamidi M.S., Gajic-Veljanoski O., Cheung A.M. (2013). Vitamin K and bone health. J. Clin. Densitom..

[8] Enokida H., Gotanda T., Oku S., Imazono Y., Kubo H., Hanada T., Suzuki S., Inomata K., Kishiye T., Tahara Y. (2002). Reversal of P-glycoprotein-mediated paclitaxel resistance by new synthetic isoprenoids in human bladder cancer cell line. Jpn. J. Cancer Res..

[9] Sidorova T.A., Nigmatov A.G., Kakpakova E.S., Stavrovskaya A.A., Gerassimova G.K., Shtil A.A., Serebryakov E.P. (2002). Effects of isoprenoid analogues of SDB-ethylenediamine on multidrug resistant tumor cells alone and in combination with chemotherapeutic drugs. J. Med. Chem..

[10] Yao X., Bai Q., Yan D., Li G., Lü C., Xu H. (2015). Solanesol protects human hepatic L02 cells from ethanol-induced oxidative injury via upregulation of *HO-1* and *Hsp70*. Toxicol. In Vitro.

[11] Roe S.J., Oldfield M.F., Geach N., Baxter A. (2013). A convergent stereocontrolled synthesis of [3-^14^C]solanesol. J. Label. Compd. Radiopharm..

[12] Taylor M.A., Fraser P.D. (2011). Solanesol: Added value from *Solanaceous* waste. Phytochemistry.

[13] Zhou H.Y., Liu C.Z. (2006). Rapid determination of solanesol in tobacco by highperformance liquid chromatography with evaporative light scattering detection following microwave-assisted extraction. J. Chromatogr. B.

[14] Fukusaki E., Takeno S., Bamba T., Okumoto H., Katto H., Kajiyami S., Kobayashi A. (2004). Biosynthetic pathway for the C45 polyprenol, solanesol, in tobacco. Biosci. Biotechnol. Biochem..

[15] Bajda A., Konopka-Postupolska D., Krzymowska M., Hennig J., Skorupinska-Tudek K., Surmacz L., Wojcik J., Matysiak Z., Chojnacki T., Skorzynska-Polit E. (2009). Role of polyisoprenoids in tobacco resistance against biotic stresses. Physiol. Plantarum..

[16] Walter M.H., Hans J., Strack D. (2002). Two distantly related genes encoding 1-deoxy-d-xylulose 5-phosphate synthases: Differential regulation in shoots and apocarotenoid-accumulating mycorrhizal roots. Plant J..

[17] Estévez J.M., Cantero A., Reindl A., Reichler S., León P. (2001). 1-Deoxy-d-xylulose-5-phosphate synthase, a limiting enzyme for plastidic isoprenoid biosynthesis in plants. J. Biol. Chem..

[18] Muñoz-Bertomeu J., Arrillaga I., Ros R., Segura J. (2006). Up-regulation of 1-deoxy-d-xylulose-5-phosphate synthase enhances production of essential oils in transgenic spike lavender. Plant Physiol..

[19] Zhang H., Niu D., Wang J., Zhang S., Yang Y., Jia H., Cui H. (2015). Engineering a platform for photosynthetic pigment, hormone and cembrane-related diterpenoid production in *Nicotiana tabacum*. Plant Cell Physiol..

[20] Carretero-Paulet L., Cairó A., Botella-Pavía P., Besumbes O., Campos N., Boronat A., Rodríguez-Concepción M. (2006). Enhanced flux through the methylerythritol 4-phosphate pathway in *Arabidopsis* plants overexpressing deoxyxylulose 5-phosphate reductoisomerase. Plant Mol. Biol..

[21] Hasunuma T., Takeno S., Hayashi S., Okumoto H., Katto H., Kajiyami S., Kobayashi A. (2008). Overexpression of 1-deoxy-Dxylulose-5-phosphate reductoisomerase gene in chloroplast contributes to increment of isoprenoid production. J. Biosci. Bioeng..

[22] Sabater-Jara A.B., Souliman-Youssef S., Novo-Uzal E., Almagro L., Belchí-Navarro S., Pedreno M.A. (2013). Biotechnological approaches to enhance the biosynthesis of ginkgolides and bilobalide in *Ginkgo biloba*. Phytochem. Rev..

[23] Hsieh M., Chang C., Hsu S., Chen J. (2008). Chloroplast localization of methylerythritol 4-phosphate pathway enzymes and regulation of mitochondrial genes in ispD and ispE albino mutants in *Arabidopsis*. Plant Mol. Biol..

[24] Kim S.M., Kuzuyama T., Chang Y.J., Kim S.U. (2006). Cloning and characterization of 2-*C*-methyl-d-erythritol 2,4-cyclodiphosphate synthase (*MECS*) gene from *Ginkgo biloba*. Plant Cell Rep..

[25] Gao S., Lin J., Liu X., Deng Z., Li Y., Sun X., Tang K. (2006). Molecular cloning, characterization and functional analysis of a 2*C*-methyl-d-erythritol 2, 4-cyclodiphosphate synthase gene from *Ginkgo biloba*. J. Biochem. Mol. Biol..

[26] Lu J., Wu W., Cao S., Zhao H., Zeng H., Lin L., Sun X., Tang K. (2008). Molecular cloning and characterization of 1-hydroxy-2-methyl-2-(*E*)-butenyl-4-diphosphate reductase gene from *Ginkgo biloba*. Mol. Biol. Rep..

[27] Kim S.M., Kuzuyama T., Kobayashi A., Sando T., Chang Y.J., Kim S.U. (2008). 1-Hydroxy-2-methyl-2-(*E*)-butenyl 4-diphosphate reductase (IDS) is encoded by multicopy genes in gymnosperms *Ginkgo biloba* and *Pinus taeda*. Planta.

[28] Berthelot K., Estevez Y., Deffieux A., Peruch F. (2012). Isopentenyl diphosphate isomerase: A checkpoint to isoprenoid biosynthesis. Biochimie.

[29] Nakamura A., Shimada H., Masuda T., Ohta H., Takamiya K. (2001). Two distinct isopentenyl diphosphate isomerases in cytosol and plastid are differentially induced by environmental stresses in tobacco. FEBS Lett..

[30] Sun J., Zhang Y.Y., Liu H., Zou Z., Zhang C.J., Li H.X., Ye Z.B. (2010). A novel cytoplasmic isopentenyl diphosphate isomerase gene from tomato (*Solanum lycopersicum*): Cloning, expression, and color complementation. Plant Mol. Biol. Rep..

[31] Block A., Fristedt R., Rogers S., Kumar J., Barnes B., Barnes J., Elowsky C.G., Wamboldt Y., Mackenzie S.A., Redding K. (2013). Functional modeling identifies paralogous solanesyl-diphosphate synthases that assemble the side chain of plastoquinone-9 in plastids. J. Biol. Chem..

[32] Hirooka K., Izumi Y., An C.I., Nakazawa Y., Fukusaki E., Kobayashi A. (2005). Functional analysis of two solanesyl diphosphate synthases from *Arabidopsis thaliana*. Biosci. Biotechnol. Biochem..

[33] Jun L., Saiki R., Tatsumi K., Nakagawa T., Kawamukai M. (2004). Identification and subcellular localization of two solanesyl diphosphate synthases from *Arabidopsis thaliana*. Plant Cell Physiol..

[34] Phatthiya A., Takahashi S., Chareonthiphakorn N., Koyama T., Wititsuwannakul D., Wititsuwannakul R. (2007). Cloning and expression of the gene encoding solanesyl diphosphate synthase from *Hevea brasiliensis*. Plant Sci..

[35] Ohara K., Sasaki K., Yazaki K. (2010). Two solanesyl diphosphate synthases with different subcellular localizations and their respective physiological roles in *Oryza sativa*. J. Exp. Bot..

[36] Jones M.O., Perez-Fons L., Robertson F.P., Bramley P.M., Fraser P.D. (2013). Functional characterization of long-chain prenyl diphosphate synthases from tomato. Biochem. J..

[37] Yan N., Zhao T., Xiang D., Gong D., Zhang H., Du Y., Liu X., Zhang Z., Liu Y. (2016). Cloning and expression analysis of solanesyl diphosphate synthase (*NtSPS*) genes in *Nicotiana tabacum*. Chin. Tob. Sci..

[38] Tamura K., Peterson D., Peterson N., Stecher G., Nei M., Kumar S. (2011). MEGA5: Molecular evolutionary genetics analysis using maximum likelihood, evolutionary distance, and maximum parsimony methods. Mol. Biol. Evol..

[39] Wang L.J., Fang X., Yang C.Q., Li J.X., Chen X.Y. (2013). Biosynthesis and regulation of secondary terpenoid metabolism in plants. Sci. Sin. Vitae.

[40] Grabherr M.G., Haas B.J., Yassour M., Levin J., Thompson D.A., Amit I., Adiconis X., Fan L., Raychowdhury R., Zeng Q. (2011). Full-length transcriptome assembly from RNA-seq data without a reference genome. Nat. Biotechnol..

[41] Mortazavi A., Williams B.A., McCue K., Schaeffer L., Wold B. (2008). Mapping and quantifying mammalian transcriptomes by RNA-Seq. Nat. Methods.

[42] Yan N., Xu X.F., Wang Z.D., Huang J.Z., Guo D.P. (2013). Interactive effects of temperature and light intensity on photosynthesis and antioxidant enzyme activity in *Zizania latifolia* Turcz. plants. Photosynthetica.

